# Supplementary material for: Epitope resurfacing on dengue virus-like particle vaccine preparation to induce broad neutralizing antibody
Source: eLife. 2018 Oct 18;7:e38970. doi: 10.7554/eLife.38970 (PMC6234032; doi:10.7554/eLife.38970)
Supplement: Supplementary file 1. [file elife-38970-supp1.docx]

Table S1. Nucleotide sequences of primers for site-directed mutagenesis used in this study

| Name | Primer sequence (5’-3’) | Amino acid substitution | Secreted |
| --- | --- | --- | --- |
| mD2VLP | ACGTGTACCACCATGGGAGAA**AAAAAA**AGAGAAAAAAGATCAGTG | His-Lys, Arg-Lys | Y |
|  | TGGGACGTGTACCACCATG**GTAGTA**AAAAAAAGAGAAAAAAGATC | Gly-Val, Glu-Val | Y |
|  | CATGGTAGTAAAAAAAAGA**TCA**AAAAGATCAGTGGCACTCG | Glu-Ser | Y |
|  |  |  |  |
| imD2VLP | AGAACATAGAAGAGAA**TCAACA**TCAGTGGCACTCG | Lys-Ser, Arg-Thr | Y |
|  |  |  |  |
| △2H2 | AGGGAAAAGTCTTCTGTTT**CCA**ACAGAGGATGGCGTGAAC | Lys-Pro | Y |
|  | CAGCAGACAAGAGAAAGGG**GAC**AGTCTTCTGTTTCCAACAG | Lys-Asp | Y |
|  | CATAGCTTGTGCAGGCGCC**GCC**CATTTAACCACACGTAAC | Phe-Ala | Y |
|  |  |  |  |
| W101G | TCCATGGTAGACAGAGGA**GGG**GGAAATGGATGTGGACTA | Trp-Gly | Y |
| N103K | GACAGAGGATGGGGAAAAGGATGTGGACTATTTGGA | Asn-Lys | N |
| G104Q | AGACAGAGGATGGGGAAATCAATGTGGACTATTTGGAAAGG | Gly-Gln | N |
|  |  |  |  |
| K307E | TCTATGTGCACAGGAAAGTTT**GAA**GTTGTGAAGGAAATAGCAGAA | Lys-Glu | N |
| K310E | ACAGGAAAGTTTAAAGTTGTG**GAG**GAAATAGCAGAAACAC | Lys-Glu | Y |
| E311R | CAGGAAAGTTTAAAGTTGTGAAG**CGA**ATAGCAGAAACACAACATGG | Glu-Arg | Y |
| E314R | AAAGTTGTGAAGGAAATAGCACGAACACAACATGGAACAATAGTT | Glu-Arg | N |
| T315H | TTGTGAAGGAAATAGCAGAA**CAC**CAACATGGAACAATAGTTAT | Thr-His | N |
| Q316P | GTGAAGGAAATAGCAGAAACA**CCA**CATGGAACAATAGTTATCAGA | Gln-Pro | Y |
| H317E | AAGGAAATAGCAGAAACACAA**GAA**GGAACAATAGTTATCAGAGTG | His-Glu | Y |
| P364R | GACAGAAAAAGATAGC**CGG**GTCAACATAGAAGCAGAACCT | Pro-Arg | N |
| W391G | GGACAACTGAAGCTCAAC**GGG**TTTAAGAAAGGAAGCAC | Trp-Gly | N |
| F392A | ACAACTGAAGCTCAACTGG**GCT**AAGAAAGGAAGCACGCTG | Phe-Ala | Y |
