# Supplementary material for: Epitope resurfacing on dengue virus-like particle vaccine preparation to induce broad neutralizing antibody
Source: eLife. 2018 Oct 18;7:e38970. doi: 10.7554/eLife.38970 (PMC6234032; doi:10.7554/eLife.38970)
Supplement: Transparent reporting form [file elife-38970-transrepform.docx]

***eLife’s* transparent reporting form**

We encourage authors to provide detailed information *within their submission* to facilitate the interpretation and replication of experiments. Authors can upload supporting documentation to indicate the use of appropriate reporting guidelines for health-related research (see [EQUATOR Network](http://www.equator-network.org/%20)), life science research (see the [BioSharing Information Resource](https://biosharing.org/)), or the [ARRIVE guidelines](http://www.plosbiology.org/article/info:doi/10.1371/journal.pbio.1000412) for reporting work involving animal research. Where applicable, authors should refer to any relevant reporting standards documents in this form.

If you have any questions, please consult our Journal Policies and/or contact us: [editorial@elifesciences.org](mailto:editorial@elifesciences.org).

**Sample-size estimation**

- You should state whether an appropriate sample size was computed when the study was being designed
- You should state the statistical method of sample size computation and any required assumptions
- If no explicit power analysis was used, you should describe how you decided what sample (replicate) size (number) to use

Please outline where this information can be found within the submission (e.g., sections or figure legends), or explain why this information doesn’t apply to your submission:

Sample size for animal study was estimated from previous similar study. The information was reported in method section.

**Replicates**

- You should report how often each experiment was performed
- You should include a definition of biological versus technical replication
- The data obtained should be provided and sufficient information should be provided to indicate the number of independent biological and/or technical replicates
- If you encountered any outliers, you should describe how these were handled
- Criteria for exclusion/inclusion of data should be clearly stated
- High-throughput sequence data should be uploaded before submission, with a private link for reviewers provided (these are available from both GEO and ArrayExpress)

Please outline where this information can be found within the submission (e.g., sections or figure legends), or explain why this information doesn’t apply to your submission:

The information on the number of samples and technical replicates is described within each figure or figure legend. Biological replications refer to numbers of mice per group.

All data presented for immunological responses of groups of mice receiving different VLP antigens are based on a representative of three independent experiments with two replicates from n=5 mice sera per group per experiment and expressed as mean + SEM. All data presented for challenge study are based on the groups of mice receiving different antigens with numbers of pulps indicated within the figure.

All animals were included for analysis. No criteria was pre-set and no randomization or blinded to the investigator test was used in this study.

In structural analyses, there were 4,217 particles were included in the final reconstruction and assessed by Fourier shell correlation (FSC) curves with the effective resolution of ~13 Å, which were enough to distinguish the key structures. The raw images, FSC were attached as supplementary materials. The 3D structure and detailed data collection information was deposited in EMDB with accession number 6926.

**Statistical reporting**

- Statistical analysis methods should be described and justified
- Raw data should be presented in figures whenever informative to do so (typically when N per group is less than 10)
- For each experiment, you should identify the statistical tests used, exact values of N, definitions of center, methods of multiple test correction, and dispersion and precision measures (e.g., mean, median, SD, SEM, confidence intervals; and, for the major substantive results, a measure of effect size (e.g., Pearson's r, Cohen's d)
- Report exact p-values wherever possible alongside the summary statistics and 95% confidence intervals. These should be reported for all key questions and not only when the p-value is less than 0.05.

Please outline where this information can be found within the submission (e.g., sections or figure legends), or explain why this information doesn’t apply to your submission:

The statistical method used in this study is described in the “Materials and Methods” section of the manuscript and. The means, SDs, and p-values are available in figure legends.

(For large datasets, or papers with a very large number of statistical tests, you may upload a single table file with tests, Ns, etc., with reference to sections in the manuscript.)

**Group allocation**

- Indicate how samples were allocated into experimental groups (in the case of clinical studies, please specify allocation to treatment method); if randomization was used, please also state if restricted randomization was applied
- Indicate if masking was used during group allocation, data collection and/or data analysis

Please outline where this information can be found within the submission (e.g., sections or figure legends), or explain why this information doesn’t apply to your submission:

Group allocation of samples was not relevant to our work.

**Additional data files (“source data”)**

- We encourage you to upload relevant additional data files, such as numerical data that are represented as a graph in a figure, or as a summary table
- Where provided, these should be in the most useful format, and they can be uploaded as “Source data” files linked to a main figure or table
- Include model definition files including the full list of parameters used
- Include code used for data analysis (e.g., R, MatLab)
- Avoid stating that data files are “available upon request”

Please indicate the figures or tables for which source data files have been provided:

Figure 4 and Figure S8 (Figure 4 and S8-source data.xlsx)

Figure 5 (Figure 5-source data.xlsx)

Figure 6 (Figure 6-source data.xlsx)

Figure 7 (Figure 7-source data.xlsx)

Figure 8 (Figure 8-source data.xlsx)
